# Time Series Modelling

**DOI:** 10.3390/e23091163

**Published:** 2021-09-04

**Authors:** Christian H. Weiß

**Affiliations:** Department of Mathematics and Statistics, Helmut Schmidt University, 22043 Hamburg, Germany; weissc@hsu-hh.de

**Keywords:** time series, models

Time series consist of data observed sequentially in time, and they are assumed to stem from an underlying stochastic process. The scope of time series approaches thus covers models for stochastic processes as well as inferential procedures for model fitting, model diagnostics, forecasting, and various other applications. While time series data have been collected for a relatively long time in history (one may recall the famous time series on sunspot numbers), the development of methods and stochastic models for such time series is more recent. Indeed, one of the motivations for announcing the Special Issue in 2020 was the fact that this year can be considered a twofold ’anniversary year’ of time series modelling. On the one hand, the correlogram, the autoregressive (AR), and the moving-average (MA) models for time series, all of which are nowadays part of any course on time series analysis and covered by any statistical software, date back to the 1920s (mainly driven by G. U. Yule, G. T. Walker, and E. E. Slutzky; see Nie and Wu [1] for a detailed discussion). On the other hand, the first comprehensive textbook on time series was published by Box and Jenkins [2] in 1970, so 2020 allowed the celebration of both the semi-centennial and centennial anniversary at the same time. In keeping with this anniversary, it was indeed possible to collect articles on a wide range of topics in this Special Issue: stochastic models for time series as well as methods for their analysis, univariate and multivariate time series, real-valued and discrete-valued time series, applications of time series methods to forecasting and statistical process control, and software implementations of methods and models for time series. The remainder of this editorial provides a brief summary of the contributions to this Special Issue, grouping the articles thematically.

Roughly one-half of the contributed articles deal with real-valued time series (thus having a continuous range). In Nono et al. [3], an entropy-based Student’s *t*-process dynamical model is proposed for dealing with non-Gaussian and non-linear univariate time series, whose relevance is demonstrated by an application to financial time series. The paper by Davidescu et al. [4] is centered around the time series of Romanian unemployment rates, which serves as the base for comparing the forecast performance of several well-established time series models. Not a single time series, but a large collection of univariate time series is considered by Lindstrom et al. [5], who use functional kernel density estimation for uncovering anomalous time series within such a collection. They apply their approaches to time series on aviation safety events as provided by the International Air Transport Association. Another data-intensive application area is electrical power forecasting, where both statistical and machine-learning methods are used. Vivas et al. [6] provide a systematic review of both types of methods (as well as of hybrid models) regarding forecast performance. Multiple time series are also considered by Sundararajan et al. [7], but now with a focus on multivariate time series having unequal dimensions. They propose and investigate a frequency-specific spectral ratio statistic, which is used to uncover differences in the spread of spectral information in a pair of such time series, and which is applied to data from stroke experiments. Another article on multivariate time series, following types of integrated vector ARMA models, is the one by Bauer and Buschmeier [8], who investigate estimators resulting from canonical variate analysis as well as novel cointegration tests. For illustration, they present an application to hourly electricity consumption data. The final article presented in the group of real-valued time series also constitutes a bridge to the next group of articles—namely, to those on discrete-valued time series. Nüßgen and Schnurr [9] consider a multivariate long-range dependent Gaussian time series, but they analyze its dependence structure based on discrete ordinal patterns derived thereof. The estimators of ordinal pattern dependence are analyzed asymptotically and within a simulation study.

The second half of contributed articles deals with time series having a discrete range, or more precisely, with count time series, where the observations are count values from the set of non-negative integers. A common approach to adapt the ARMA model known for real-valued time series to the case of count time series consists of substituting the multiplications within the ARMA recursive model by types of thinning operators; see Chapters 2–3 in Weiß [10] for detailed background. The resulting integer-valued counterparts to the ordinary AR and MA models are then referred to as INAR and INMA models, respectively. In Huang and Zhu [11], a new type of the classical INAR model using binomial thinning is proposed, where the innovations follow the one-parameter Bell distribution. Stochastic properties and estimation approaches are investigated, and applications to time series consisting of crime counts and strike counts are presented. Liu and Zhu [12], by contrast, develop an extension of the INAR model, where a new type of thinning operator is used, relying on the extended binomial distribution. The resulting model is able to flexibly adapt to different types of dispersion behavior, which is also demonstrated by several real-data examples. Furthermore, Yu and Wang [13] consider an extension of the binomial thinning operator, achieved by allowing for a dependent counting series, and this time, the operator is used within the class of INMA models. Properties of, and estimation for, this new type of INMA model are investigated, and they are illustrated by an application to a crime-counts time series. While the three aforementioned articles consider stationary and linear count time series, the contribution by Liu et al. [14] deals with non-stationary and non-linear time series as obtained from the periodic self-exciting threshold INAR model. Properties and estimation are discussed, and an application to monthly counts of claimants is presented. In Li et al. [15], again an INAR model is considered (using a randomized binomial thinning operator); however, now the main focus is not on the model itself, but on an approach for statistical process control. The authors use a cumulative sum chart for process monitoring, discuss its performance evaluation, and apply it to a crime-counts time series. Contrary to the aforementioned papers, the articles by Kim et al. [16] and Shapovalova et al. [17] refer to multivariate count time series. For a bivariate count time series following an integer-valued generalized AR conditional heteroscedastic (INGARCH) model, Kim et al. [16] propose a minimum density power divergence estimator being robust against outliers. The asymptotics of the estimator are investigated, and an application to bivariate crime counts is presented. Shapovalova et al. [17] consider two types of models for multivariate count time series: a log-linear multivariate INGARCH model and a non-linear state-space model. These models serve as a base for a forecast performance comparison. As real-world applications, count time series about bank failures and transactions are used. Last but not least, Stapper [18] developed a comprehensive software package (in the Julia language) for count time series modelling. The package fits different types of INARMA and INGARCH models, and it offers functions for model diagnostics, forecasting, etc. In his paper, Stapper [18] illustrates the application and the potential of “CountTimeSeries.jl” with several real-data examples and simulation experiments.
